# Virtual Reality in Health Care: Bibliometric Analysis

**DOI:** 10.2196/32721

**Published:** 2021-12-01

**Authors:** Christian Matthias Pawassar, Victor Tiberius

**Affiliations:** 1 Faculty of Economics and Social Sciences University of Potsdam Potsdam Germany

**Keywords:** virtual reality, healthcare, bibliometric analysis, literature review, citation analysis, VR, usability, review, health care

## Abstract

**Background:**

Research into the application of virtual reality technology in the health care sector has rapidly increased, resulting in a large body of research that is difficult to keep up with.

**Objective:**

We will provide an overview of the annual publication numbers in this field and the most productive and influential countries, journals, and authors, as well as the most used, most co-occurring, and most recent keywords.

**Methods:**

Based on a data set of 356 publications and 20,363 citations derived from Web of Science, we conducted a bibliometric analysis using BibExcel, HistCite, and VOSviewer.

**Results:**

The strongest growth in publications occurred in 2020, accounting for 29.49% of all publications so far. The most productive countries are the United States, the United Kingdom, and Spain; the most influential countries are the United States, Canada, and the United Kingdom. The most productive journals are the Journal of Medical Internet Research (JMIR), JMIR Serious Games, and the Games for Health Journal; the most influential journals are Patient Education and Counselling, Medical Education, and Quality of Life Research. The most productive authors are Riva, del Piccolo, and Schwebel; the most influential authors are Finset, del Piccolo, and Eide. The most frequently occurring keywords other than “virtual” and “reality” are “training,” “trial,” and “patients.” The most relevant research themes are communication, education, and novel treatments; the most recent research trends are fitness and exergames.

**Conclusions:**

The analysis shows that the field has left its infant state and its specialization is advancing, with a clear focus on patient usability.

## Introduction

There is no definite date for the first virtual reality (VR) application but the Sensorama device described by Morton Heilig in 1955 can be seen as a possible starting point [1,2]. His invention is one of the earliest known examples of immersive technology incorporating vision, sound, smell, as well as the sensation of touch, thereby letting users experience an illusory form of reality [2,3]. VR can be defined as a technology that makes users believe that they are in another place, based on heavily influencing the primary sensory inputs with computer-generated data [4]. What started as a pure form of entertainment involving immersion and interactivity has now become a thriving market with a wide range of use cases including recreation, communication, research, education, and health care [5-7]. VR initially started out as a luxury but has recently become more accessible, with companies like Facebook, Google, Samsung, and Sony making large investments in this technology [8,9]. The health sector benefits from this development as VR technology for entertainment purposes can also improve medical training [5,10,11], leading to a fusion of gaming and educational training called serious gaming [12]. Professionals, researchers, and students get early exposure to equipment, procedures, and clinical settings, as well as feedback, without putting anyone at risk [5,11]. The relevance of VR in the health sector is also increasing due to breakthroughs such as O’Keefe and Moser’s discovery of the brain’s “GPS” (for which they received the Nobel Prize), highlighting the possibilities of VR-supported studies [13]. While analyzing the brain activity of rodents in a VR setting, these researchers found “cells that constitute a positioning system in the brain” [14,15]. Additionally, a wide array of VR engines and VR applications for experimental and computational use is becoming more accessible for everyone [14,16-18]. Lastly, VR is a driving factor in the development of new treatments or the revamping of older ones [19,20]. Novel approaches include phobia therapy [21], rehabilitation [22], and many more. These new forms of psychological and physical treatment help patients and health professionals to achieve better results in care, healing, and comfort [19,20,23,24].

The widespread use of VR makes it an increasingly relevant topic for research, leading to a growing body of scholarly literature in recent years [25], which has become more and more complex and fragmented. As a consequence, a systematic overview of research on VR in health care is needed. Our research goal is to provide such a comprehensive overview, based on a bibliometric analysis. We aim to identify current trends to promote and guide future research.

Our study supplements previous bibliometric analyses on VR in the health sector, which have a narrower focus on specific use cases, such as dementia [26], autism [27], or rehabilitation [28]. Other bibliometric analyses are outdated and do not adequately represent the current state of available material [29]. Some bibliometric analyses are not limited to VR but also include augmented reality (AR) [25,30]. Cipresso et al [31] provided a large-scale network and cluster analysis for both VR and AR across all scientific disciplines. In contrast to all these analyses, this study aims to provide a current and comprehensive overview of VR research in the health sector via a bibliometric analysis. It therefore contributes to research on VR in health care by measuring and mapping the body of literature on this topic.

## Methods

### Search and Screening Strategy

We conducted a title search for “virtual reality” or “VR” on the Web of Science Core Collection on April 19, 2021. The title search ensured that only publications focused on VR were included in the data set, while publications touching on VR as a side aspect were excluded. The search yielded 12,979 publications from 1994-2021.

**Figure 1:**
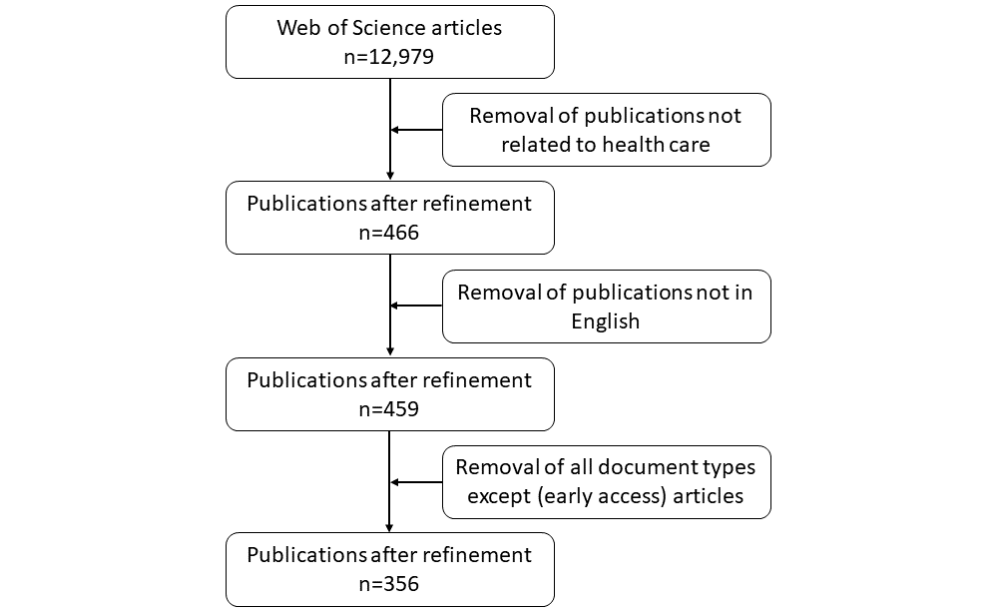
Data collection process.

The data set was narrowed down by focusing on publications written in the predominant scientific language (English). We selected only publications in health care–related categories: Health Care Science Services, Public Environmental Occupational Health, Health Policy Services, and Primary Health Care. Finally, we excluded all document types other than articles (“early access” articles were included). This elimination process helped guarantee that only peer-reviewed research was included in the bibliometric analysis. The final data set consists of a total of 356 publications (Figure 1).

### Bibliometric Analysis

Bibliometrics focuses on the analysis of quantifiable publication data [32], which can be used to create objective and reproducible results [33] and offer insights into relationships between analyzed documents [34]. The basic measures for bibliometric performance analyses are the number of articles (representing productivity) and the number of citations (representing impact or influence) [35]. We focused on performance data regarding productivity and impact by year as well as by country, journal, and author. Frequencies and pro rata percentages in each category were based on publication years, total share of records, or total global citation scores. Country scores were based on the affiliation data provided by the authors themselves. Additionally, the most used, most co-occurring, and most recent keywords were examined to find and structure the main research themes. Furthermore, the twenty most important keywords were determined. These bibliometric indicators are regularly used to provide a bibliometric overview of a field [36] and to determine the most influential countries, authors, and journals of a field [37]. Based on bibliometric findings, future research trends can be derived [37].

This analysis involved the use of total global citation scores and a computation of popularity based on appearance frequency. The underlying data for the bibliometric analysis was analyzed with BibExcel (version 2016-02-20), HistCite (version 12.03.17), and VOSviewer (version 1.6.16).

## Results

### Annual Productivity

Table 1 depicts the annual numbers of publications on VR in health care from 1994 to April 2021. From 1994 to 2020, the average growth rate of scientific publications about VR in health care was 20.46%. Including the partial year 2021, the calculated average growth rate is currently 13.98%. The average growth rate from 1994-2010 was 10.58%, which increased to 20.54% and 60.05% for 2011-2015 and 2016-2020, respectively. The growth rate from 2016-2021 is currently 13.40%. There was a surge of scientific publications starting around 2014, accounting for 80.62% of all eligible articles. The biggest increase was in 2020, accounting for 29.49% of all analyzed scholarly works.

Overall, 88.8% (315/356) of the analyzed scientific articles were published by researchers from 20 countries (Table 1). The top three countries are the United States (94/356, 26.4%), the United Kingdom (28/356, 7.9%), and Spain (24/356, 6.7%). Another 24 countries not shown in Table 2 contributed to VR research in health care. The percentages shown in Table 1 relate to the total number of publications by the depicted countries (N=315), not the overall data set.

The country with the highest global citation score is the United States, accounting for nearly one-third of all citations in the data set (1995/6701, 29.8%). Canada is ranked second, with about one-tenth of all citations (659/6701, 9.8%), followed by the United Kingdom in third place, with about 9% of all citations (613/6701, 9.1%). The percentages in Table 3 refer to the total number of citations of the top 20 countries, not the complete data set.

**Table 1:** Country productivity and country impact.

Year	Number of publications
1994	1
1996	1
1997	1
2000	3
2001	1
2002	1
2003	2
2004	3
2005	4
2006	1
2007	4
2008	5
2009	8
2010	5
201	9
2012	10
2013	8
2014	15
2015	19
2016	16
2017	28
2018	31
2019	43
2020	105
2021	30
Early access	2

**Table 2:** Country productivity (N=315).

Rank	Country	Output, n (%)
1	United States of America	94 (29.8)
2	United Kingdom	28 (8.9)
3	Spain	24 (7.6)
4	Canada	21 (6.7)
5	People’s Republic of China	18 (5.7)
6	Australia	16 (5.1)
6	Italy	16 (5.1)
7	South Korea	15 (4.8)
8	Germany	13 (4.1)
8	Taiwan	13 (4.1)
9	Netherlands	12 (3.8)
10	France	8 (2.5)
11	Brazil	6 (1.9)
12	Finland	5 (1.6)
12	Israel	5 (1.6)
12	Turkey	5 (1.6)
13	Norway	4 (1.3)
13	Poland	4 (1.3)
13	Portugal	4 (1.3)
23	Sweden	4 (1.3)

**Table 3:** Country impact (N=6432).

Rank	Country	Output, n (%)
1	United States	1995 (31.0)
2	Canada	659 (10.3)
3	United Kingdom	613 (9.5)
4	Italy	493 (7.7)
5	Netherlands	383 (6.0)
6	Norway	362 (5.6)
7	Germany	338 (5.3)
8	Australia	303 (4.7)
9	Spain	267 (4.2)
10	Switzerland	256 (4.0)
11	Belgium	147 (2.3)
12	Israel	112 (1.7)
13	Taiwan	100 (1.6)
14	People’s Republic of China	98 (1.5)
15	Singapore	63 (1.0)
16	France	60 (0.9)
17	Mexico	58 (0.9)
18	New Zealand	47 (0.7)
19	India	43 (0.7)
20	Finland	35 (0.5)

### Journal Productivity and Impact

The highest-ranking journal regarding productivity is the Journal of Medical Internet Research, with 34 of the 356 total publications (9.6%). In second place is JMIR Serious Games (29/356, 8.2%), followed by the Games for Health Journal in third position (28/356, 7.9%). The percentages in Table 3 relate to the total number of articles in the top 20 most productive journals; the articles in the full data set were published in 70 additional journals not shown in Table 4.

The most cited journal is Patient Education and Counselling, with a total global citation score of 428 of the 3993 citations in the entire data set (10.7%). In second place is Medical Education (312/3993, 7.8%), followed by Quality of Life Research (281/3993, 7%). Again, out of the 90 journals included in the data set, 70 are not shown in Table 5. The percentages given in the table relate to the 3105 citations received by the top 20 journals, not the whole data set.

The three authors with the highest productivity are Riva (9/356, 3%), del Piccolo (6/356, 2%), and Schwebel (6/356, 2%). Out of 1698 authors in the data set, 1678 are not shown in Table 6. Most authors (1532, 90.2%) only published one article on VR in health care, while 129 (7.6%) published two qualifying articles.

The author with the highest impact as determined by overall citations is Finset, with a total of 340 citations of 20,363 (1.67%) in the whole data set. The next most influential authors are del Piccolo and Eide, both in second place with 308 citations (308/20,363, 1.51%). Taking all citations and authors into account, the average is 20.36 citations per researcher. The computed h-index of an author (shown in Table 7) relates to the subject of this bibliometric analysis, and does not include other publications by the author. Once again, the displayed percentage relates to the total number of citations represented in the table, not the whole data set.

**Table 4:** Journal productivity (N=235).

Rank	Journal	Output, n (%)
1	Journal of Medical Internet Research	34 (14.5)
2	JMIR Serious Games	29 (12.3)
3	Games for Health Journal	28 (11.9)
4	International Journal of Environmental Research and Public Health	27 (11.5)
5	Patient Education and Counselling	16 (6.8)
6	Simulation in Healthcare: The Journal of the Society for Simulation in Healthcare	11 (4.7)
7	Journal of Healthcare Engineering	10 (4.3)
8	Technology and Healthcare	10 (4.3)
9	Accident Analysis and Prevention	8 (3.4)
9	Annual Review of Cybertherapy and Telemedicine 2015: Virtual Reality in Healthcare: Medical Simulation and Experiential Interface	8 (3.4)
10	Annual Review of Cybertherapy and Telemedicine 2014: Positive Change: Connecting the Virtual and the Real	7 (3.0)
11	Frontiers in Public Health	6 (2.6)
11	Journal of Medical Systems	6 (2.6)
12	Aerospace Medicine and Human Performance	5 (2.1)
12	Aviation Space and Environmental Medicine	5 (2.1)
12	Injury Prevention	5 (2.1)
12	International Journal of Occupational Safety and Ergonomics	5 (2.1)
12	JMIR Research Protocols	5 (2.1)
12	Methods of Information in Medicine	5 (2.1)
12	Work: A Journal of Prevention Assessment & Rehabilitation	5 (2.1)

**Table 5:** Journal impact (N=3105).

Rank	Journal	Citations, n (%)
1	Patient Education and Counselling	428 (13.8)
2	Medical Education	312 (10.0)
3	Quality of Life Research	281 (9.1)
4	Aviation Space and Environmental Medicine	247 (8.0)
5	Games for Health Journal	234 (7.5)
6	Academic Medicine	213 (6.9)
7	Accident Analysis and Prevention	196 (6.3)
8	Journal of Medical Internet Research	142 (4.6)
9	Nicotine & Tobacco Research	111 (3.6)
10	Supportive Care in Cancer	106 (3.4)
11	Telemedicine Journal and e-Health	101 (3.3)
12	Methods of Information in Medicine	100 (3.2)
13	Simulation in Healthcare: The Journal of the Society for Simulation in Healthcare	90 (2.9)
14	International Journal on Disability and Human Development	89 (2.9)
15	Journal of Medical Systems	80 (2.6)
15	Simulation in Healthcare	80 (2.6)
16	Psychology & Health	75 (2.4)
16	Technology and Health Care	75 (2.4)
17	International Journal of Medical Informatics	74 (2.4)
18	JMIR Serious Games	71 (2.3)

**Table 6:** Author productivity (N=87).

Rank	Author	Publications, n (%)
1	G Riva	9 (10)
2	L del Piccolo	6 (7)
2	DC Schwebel	6 (7)
3	A Finset	5 (6)
3	J Gutierrez-Maldonado	5 (6)
3	G Humphris	5 (6)
3	BK Wiederhold	5 (6)
3	SC Yeh	5 (6)
4	H Eide	4 (5)
4	M Ferrer-Garcia	4 (5)
4	L Smith	4 (5)
4	MD Wiederhold	4 (5)
4	C Zimmermann	4 (5)
5	SAW Andersen	3 (3)
5	WP Brinkman	3 (3)
5	R Cano de la Cuerda	3 (3)
5	P Cipresso	3 (3)
5	A Fisher	3 (3)
5	I Fletcher	3 (3)
5	C Goss	3 (3)

**Table 7:** Author impact (N=5459).

Rank	Author	Citations, n (%)	H-index
1	A Finset	340 (6.2)	5
2	L del Piccolo	308 (5.6)	5
2	H Eide	308 (5.6)	4
3	G Humphris	300 (5.5)	5
4	C Zimmermann	297 (5.4)	4
5	W Rogers	281 (5.2)	3
6	M Rimondini	273 (5.0)	3
7	C Goss	273 (5.0)	3
8	I Fletcher	271 (5.0)	3
9	YM Kim	256 (4.7)	2
9	S Bergvik	256 (4.7)	2
9	P Salmon	256 (4.7)	2
9	J Bensing	256 (4.7)	2
9	C Heaven	256 (4.7)	2
9	L Zandbelt	256 (4.7)	2
9	W Langewitz	256 (4.7)	2
9	S van Dulmen	256 (4.7)	2
9	H de Haes	256 (4.7)	2
10	L Wissow	256 (4.7)	2
11	S Qian	248 (4.5)	2

### Most Used and Co-Occurring Keywords

As shown in Table 8, the most used keyword is “virtual” (322/356, 90.4%), followed by “reality” (321/356, 90.2%). The third most used keyword is “training” (60/356, 16.9%).

**Figure 2:**
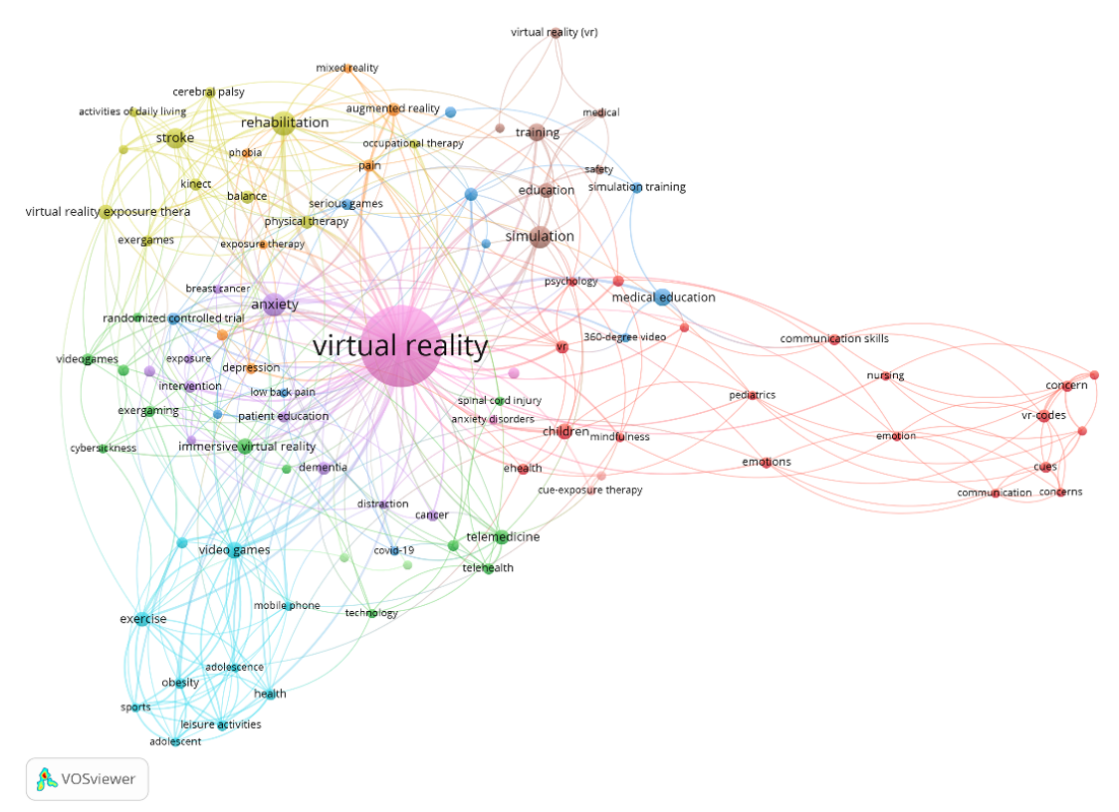
Keyword co-occurrence map.

The author keyword co-occurrence analysis led to 11 clusters, with a minimum citation threshold of 3 and 94 qualifying keywords. Figure 2 depicts the keyword co-occurrence map generated by VOSviewer. The 11 clusters are distinguished by different colors. The clusters comprise keywords, which are often mentioned together in the keyword lists of the articles in the literature sample and therefore have the tendency to form groups that represent common research themes. Of these clusters, 3 are relatively small, with only 2-3 keywords. Half of the clusters are made up of 10 or more keywords. Being presented as a cluster does not necessarily mean that publications deal with the same topic, albeit all qualifying publications within a respective cluster complement each other. Furthermore, 3 clusters have an everyday focus, 3 other ones address the specifics of VR training, 4 have diverse health care–related fields as their common topic, and one is centered around known issues in VR settings. The clusters are described in greater detail below.

**Table 8:** Most used keywords.

Rank	Keyword	Occurrences (N=1168), n (%)	Overall percentage of occurrences (n=356)
1	Virtual	322 (27.6)	90.4
2	Reality	321 (27.5)	90.2
3	Training	60 (5.1)	16.9
4	Based	55 (4.7)	15.4
5	Using	51 (4.4)	14.3
6	Trial	36 (3.1)	10.1
7	Randomized	32 (2.7)	9.0
8	Patients	30 (2.6)	8.4
9	Therapy	29 (2.5)	8.1
10	Simulation	28 (2.4)	7.9
11	Controlled	26 (2.2)	7.3
12	Health	24 (2.0)	6.7
13	Children	23 (2.0)	6.5
14	Pilot	22 (1.9)	6.2
15	Rehabilitation	20 (1.7)	5.6
16	Effects	19 (1.6)	5.3
16	Immersive	19 (1.6)	5.3
17	Care	17 (1.5)	4.8
17	Effect	17 (1.5)	4.8
27	Treatment	17 (1.5)	4.8

### Clusters

#### Cluster 1: Communication, Especially in Pediatrics

This large cluster covers 19 keywords revolving around promoting a more rounded caretaker/patient dialogue, with a focus on children. Some papers address issues regarding hindered communication with children and how VR can help with that [38,39], as well as the development of communication skills for patients [40,41] and medical staff [42,43].

#### Cluster 2: On-site or Telemedicine Health Care

This cluster containing 12 items highlights some positive aspects in caretaking via VR. A rather large focus lies with the enrichment and possibilities of interventions and care from a distance offering new and effective methods [21,44,45]. A smaller portion sees VR in use for on-site interventions [46].

#### Cluster 3: Physical Health Training

This cluster with 12 items specifies similarities and discrepancies between fitness training in real-life and VR settings. Findings of articles within this cluster are that VR-based training is effective but not more effective than real-life trainings [47,48]. VR-supported physical exercise can be applied to both private fitness endeavors and physical therapy, as the next cluster shows.

#### Cluster 4: Physical Rehabilitation

This group of keywords containing 11 items addresses the flexibility of VR in addition to conventional rehabilitation programs [49,50], and the possibility of expanding the reach of treatments beyond clinically controlled settings to achieve better results [22]. In addition, this cluster includes the adaption of VR games detached from any clinical setting and found the VR games had a positive effect on patient rehabilitation comparable to trainings conducted by medical professionals [51,52].

#### Cluster 5: Geriatric Care

This section containing 10 items centers around the care of older adults. VR was used to tackle dementia and memory loss [53-55]. Other application areas include VR interventions in life-threatening situations to improve patients’ moods [56], as well as patient education and training.

#### Cluster 6: Motivation, Health, and Adolescents

This cluster, also containing 10 items, addresses the need to motivate adolescents to partake in physical activities to tackle motivational and health issues. The focus is on cultivating fascination and enjoyment in young adults regarding the possibilities of VR and educating parents to promote the consideration of VR where antipathy might be prevalent [57-59]. This cluster underscores the need for awareness training.

#### Cluster 7: Psychological Treatments

This 7-item cluster focuses on new treatment possibilities, particularly for depression and stress disorders. Topics range from posttraumatic experience and chronic disorder treatments to preventive strategies [60-62], increasing the need for more training about the increased options for effective handling.

#### Cluster 8: Accident Prevention

This group of 7 keywords includes ophthalmology research and implements eye-tracking features to analyze behavior in dangerous situations. This is feasible because VR headsets are often very close to the eyes, and already analyze how and where the user moves in a predefined space [63-65], offering a safe environment for researchers to analyze test subjects in otherwise unworkable experimental situations.

#### Cluster 9: Palliative Care

This rather small cluster comprises only 3 keywords. The articles in this cluster focus on anxiety in people with terminal diseases and how to broaden the range of effective treatments [66]. It is closely related to cluster 7, with a focus on providing relief for the patient. For effective use, more training for caretakers needs to take place.

#### Cluster 10: Everyday Health Support

This small cluster with 2 keywords promotes interventions in relation to binge eating [67,68], but future fields of application include other interventions for working toward a healthier life every day via small reminders.

#### Cluster 11: Aftereffects of VR Environments

The last cluster also includes 2 keywords and addresses issues that may occur when people experience or cease using VR [69,70]. This is relevant for everyday life as well as trainings to increase awareness.

**Figure 3:**
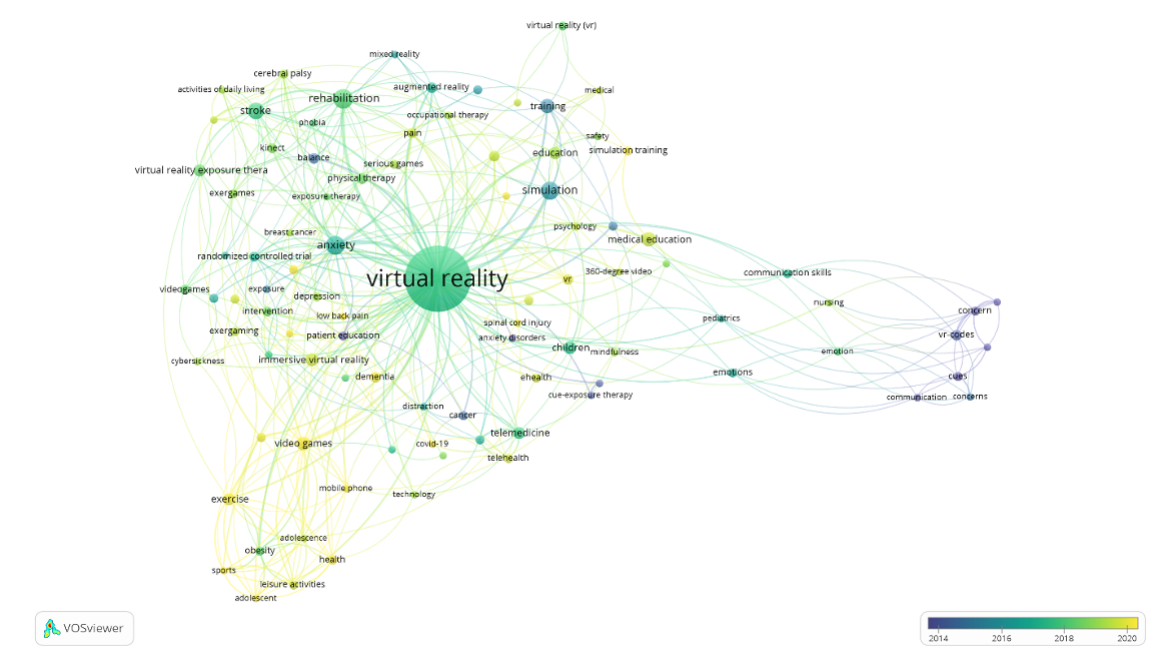
Keyword trend from 2013-2021.

Figure 3 depicts the trend evolution of keywords. The map shows how the importance of keywords changed for VR in health care starting around 2013-2014 (blue) to the current date (yellow). Earlier focal points were about concerns with the new technology and possible fields of use. A selection of representative keywords is “concern,” “communication,” “anxiety disorders,” and “patient education.” A few years later, these application fields broadened and started to include medicine-related trends. This is seen in keywords such as “telemedicine,” “physical therapy,” “exposure therapy,” and “randomized controlled trial.” The latest trend sees VR in health care become centered around education and exergames, so-called exercise games for mental and physical health, as well as mobility for virtual realities. Representative keywords are “sport(s),” “video games,” “simulation training,” and “mobile phone.”

## Discussion

### Overview

In this study, research focusing on VR in health care from 1994 to 2021 was analyzed by bibliometric measures. The focal points for this analysis were publication and author scores, as well as a trend analysis. Overall, research has been steadily increasing, with a recent spike in publications, underlining the relevance of research on VR applications in health care.

### Growth Rate of Publications

Although VR emerged in the 20th century and has been a topic in health care–related research for over 25 years, it was initially a niche topic [2]. An increase in the number of VR publications can be seen between 2015 and 2017, with a notable spike in 2020. The growth rate nearly tripled to 60.05% when compared to the previous 5 years (20.54%). What may have been a decisive factor in this change was the sudden increase in investments and availability of high-quality technology, which increased scientific interest and use [8]. The gaming industry had a major impact, showcasing the manifold (though mostly unintentional) benefits and possibilities via development of new headsets and programs, which could be appropriated and modified for new applications [11,14,17]. Since then, an increasing number of medical fields saw the potential of VR for data collection, treatments, or trainings [10,41,65]. Following the past growth pattern of health care–related VR research, it can be expected that this field will continue to grow.

### Publication Pattern

VR research is being conducted around the globe, with over one-fifth of all countries contributing to scientific progress in this field. The United States, as in many other fields, tends to lead research in this field. The top countries are consistently similar across different rankings, at most just swapping placements [71,72]. Germany, Spain, and the United Kingdom are also driving forces in the development of other digital health interventions such as telemedicine and artificial intelligence [72,73]. However, there is a notable absence of emerging countries, which could reap the benefits of the digitalization of their health sectors [74], especially as VR technology becomes more affordable [9]. The recent positive VR market development could help these countries to overcome obstacles including funding, distribution prioritization, and language support, promoting a future shift in research hotspots and more exponential growth in publications [75]. After all, research on VR and related topics of digitalization could have a large combined positive effect for developed and developing countries, which may result in cooperation and dialogue toward future progress.

The journals publishing VR-related health care content are mostly high-impact and well-known journals but lower-impact journals also add to the increasing reach and availability of research. With an average of 5 authors per publication (1698/256), VR in health care has seen growth in co-authorship over time. In terms of citations, the high-impact journals take the lead.

Scientists’ productivity and impact do not always go hand in hand. Due to the novelty and rapid development of the field, well-established scientists or articles do not exist yet. This could change over time, as rapid growth based on novelty is usually finite.

### Trends and Research Themes

After clustering the author keywords, clear distinctions were apparent, with the currently highest trending area of research involving communication and the integration of VR for educational purposes. This topic spans over manifold instances like patient and caretaker interactions, communication training, and new forms of education [10,38,43]. The second most prominent field combines telemedicine and VR, with a foray into the development of VR-based “medical professionals.” The focus is on the implementation of a VR setting in which health care professionals do not necessarily need to be physically present, or even involved in the procedure at all [45,46]. This also includes the development of new treatments using VR, such as for phobias and mental disorders [21,61]. The third most important area is centered around physical activities for both younger and older people [47,59]. The findings here see great potential and marketing opportunities for a healthy lifestyle and better life expectancy by using VR.

When looking at the prominence of keywords from 2013 to 2021, the ranking is reversed. Modern research focuses on the fitness and health opportunities of VR and on (smaller) interventions to promote better health behavior, while the communication-related and educational aspects of VR in health care tend to be less researched. In particular, the surge of interest in this topic started at the end of 2019, which could be directly related to the COVID-19 outbreak, during which VR fitness may have been one of the few viable options for implementing physical activity into daily life. Another reason for the reversed relevance of keywords might be the latest developments in VR technology, such as omnidirectional treadmills, optimized gaming gear, and newly released games using new technologies that represent untapped research potential for fitness and entertainment [76,77].

Health care–related VR research has seen strong growth in the past decades. Current contexts and possibilities underline the potential of this field to gain more traction in the near future. The cross-sectional methods for implementation and simple ways of integrating VR into current systems are what makes the technology so interesting and worth researching. As a field, VR has passed the early first steps of development and is no longer under the radar. It can be expected that, with further technological progress, the availability of VR will increase, so that emerging countries can increasingly benefit from this technology.

However, VR in health care also has some obstacles, such as motion sickness, a lost sense of presence, eye strain, or inappropriate responses in the real world [78,79]. Future research can be expected to focus on how to tackle these challenges. In particular, holographic projections have the potential to alleviate many of the negative symptoms of VR [80]. Another issue is telemedicine, which is currently limited to algorithms or an AI responding to medical personnel or patients [42], rather than focusing on VR. Future integrations could see cybernetics become an essential part of VR to probe into autonomous health care with the help of robotics [81]. Even though this raises many questions and involves barriers [82], it could become a necessity to tackle the problem of a lack of health care professionals who can take care of the increasingly aging population [83]. Older adults’ acceptance of technology can also be challenging in this context. However, recent research seems to suggest these claims may be unfounded [83]. Future research could therefore provide guidelines to increase or secure older patients’ acceptance. Only a few standardized guidelines have been developed so far [84].

### Limitations

This bibliometric analysis has some limitations. First, our search was limited to the Web of Science, which is a widely used academic database. However, the use of other databases, such as Scopus or Google Scholar, may have provided slightly different results. Second, our analysis only included articles published in English, the lingua franca of science. The inclusion of other languages, grey literature, and books might also have led to different outcomes, especially as scholars from different cultures might have different perspectives on the use of VR in health care. Third, searching article titles alone and using only two search terms was very limiting. As our goal was to focus on literature that deals with VR as the main research theme, a title search was more appropriate than a topic search. However, articles dealing with VR as a side aspect might also enrich the body of knowledge in the field. Therefore, we encourage future research to also use topic searches including abstracts and keywords, and extend the range of search terms. Fourth, the inclusion of the specified disciplines alone might have excluded relevant articles that have been published in more technologically oriented journals rather than heath care–related ones. Future research might therefore use “health*” as an additional search term rather than using a filter based on disciplines. Fifth, whereas publication and citation numbers are objective, the interpretation of keyword clusters has a subjective character; other researchers may have drawn different conclusions.

An area which should also be pursued in future studies, which has not seen enough attention thus far, is the possible interconnection between AR and VR, which are closely related. However, mix-ups between these two occur, and AR might be as feasible for use in health care as VR. In addition, they might offer benefits upon their combined use, which should be analyzed further.

### Conclusion

This bibliometric analysis aimed to give an overview of VR-related research in health care. It comprises 356 publications across about 27 years, from 1994 to 2021 (partial year). The main results are the following: (1) VR-related publications in health care have seen increased growth, (2) developed countries are the driving force in health care–related VR research but the topic has already been researched around the world, (3) the three predominant research themes center around communication, education, and novel treatments, and (4) the most recent research trends cover fitness and exergames for VR in health care. The analysis shows that the field has left its infant state and the research is becoming increasingly specialized, with a clear focus on patient usability. Future research should broaden the range of involved countries, industries, and companies.

## Abbreviations

AR: augmented reality
VR: virtual reality
